# Tyrosine Kinase Inhibitors Could Be Effective Against Non-small Cell Lung Cancer Brain Metastases Harboring Uncommon *EGFR* Mutations

**DOI:** 10.3389/fonc.2020.00224

**Published:** 2020-03-05

**Authors:** Chunhua Ma, Juncheng Zhang, Dongjiang Tang, Xin Ye, Jing Li, Ning Mu, Zhi Li, Renzhong Liu, Liang Xiang, Chuoji Huang, Rong Jiang

**Affiliations:** ^1^Tianjin Key Laboratory of Cerebral Vascular and Neurodegenerative Disease, Department of Intervention, Tianjin HuanHu Hospital, Tianjin, China; ^2^Zhuhai SanMed Biotech Ltd., Zhuhai, China; ^3^Joint Research Center of Liquid Biopsy in Guangdong, Hong Kong and Macao, Zhuhai, China; ^4^Zhuhai Livzon Gene Diagnostics Ltd., Zhuhai, China

**Keywords:** non-small cell lung cancer, brain metastasis, tyrosine kinase inhibitors, epidermal growth factor receptor, mutation

## Abstract

**Background:** The significance of uncommon *epidermal growth factor receptor* (*EGFR*) mutations in patients with non-small cell lung cancer (NSCLC) and brain metastasis (BM) remains unclear. Cerebrospinal fluid (CSF) liquid biopsy is a novel tool for assessing *EGFR* mutations in BM. This study aimed to evaluate the *EGFR* mutations in patients with NSCLC and newly diagnosed BM and to examine the effect of EGFR tyrosine kinase inhibitors (TKI) on BM harboring CSF-tested uncommon *EGFR* mutations.

**Methods:** This was a prospective study of 21 patients with NSCLC and BM diagnosed between 04/2018 and 01/2019. CSF was obtained to detect the BM *EGFR* mutations by next-generation sequencing. BM characteristics at magnetic resonance imaging (MRI) and EGFR-TKI response were examined.

**Results:** Of 21 patients with NSCLC, 10 (47.6%) had leptomeningeal metastasis (LM), while 11 (52.4%) had brain parenchymal metastasis (BPM); 13 (61.9%) had confirmed *EGFR* mutation-positive primary tumors. The uncommon mutation rate in CSF ctDNA was 33.3% (7/21). Among those with *EGFR* mutation-positive primary tumors, the rate of uncommon *EGFR* mutations in CSF was 53.8% (7/13). Uncommon *EGFR* mutations were more common in patients with LM than in patients with PBM (6/11, 54.5% vs. 1/10, 10%), and included G719A, L861Q, L703P, and G575R. TKI was effective for four patients with BMs harboring uncommon *EGFR* mutations.

**Conclusion:** In patients with NSCLC and LM, the rate of uncommon *EGFR* mutation was high. The BMs with uncommon *EGFR* mutations seem to respond to EGFR-TKI treatment. CSF liquid biopsy could reveal the *EGFR* genetic profile of the BM and help guide treatment using small-molecule TKI.

## Background

Brain metastases (BM) occurs in 30–50% of patients with non-small cell lung cancer (NSCLC) during the course of their disease (1). About 50% of the BMs are diagnosed at presentation of NSCLC, with 50–60% as the only site of distant metastasis (1). Patients with NSCLC and BMs have a poor prognosis, and the median survival is only 1–2 months (2, 3). BMs include parenchymal BMs (PBMs) and leptomeningeal metastases (LMs). LMs are less common than PBMs, with an occurrence rate of 3.4–3.8% in NSCLC, but their prognosis is worse (4, 5).

The management of BMs from NSCLC mostly includes surgery and radiation therapy; chemotherapy is seldom applied, and targeted drugs could be more effective than chemotherapy (6). In NSCLC, the targeted therapies mainly include tyrosine kinase inhibitors (TKI). TKIs have replaced chemotherapy because of better responses and survival rates (7–9). Recently developed EGFR-TKIs, e.g., osimertinib, specifically address the challenges of acquired drug resistance and low blood-brain barrier (BBB) permeability of first and second-generation TKIs, demonstrating efficacy in the CNS (10). Nevertheless, only NSCLC cells harboring *epidermal growth factor receptor* (*EGFR*) sensitizing mutations will respond to EGFR TKIs (1). Activating mutations in *EGFR* are found in 20–40% of NSCLC, with exon 19 deletions (45%) and exon 21 L858R mutations (40–45%) as the most common mutations (10). In NSCLC patients with BMs, the prevalence of *EGFR* mutations has been reported to be 39–63% in Asians (11, 12) and 2–40% in North American and European populations (13, 14). A retrospective study in China showed that the rate of uncommon mutations [i.e., mutations other than 19Del and L858R (15)] was high, with 12% of 1,837 Chinese patients with NSCLC *EGFR* mutations having non-classical mutations such as exon 20 insertion (30%), G719X mutation (21%), L858R complex mutation (17%; complex mutation defined as more than one *EGFR* mutation within a tumor sample) and T790M complex mutation (14%) (16). Importantly, different *EGFR* mutations respond differently to TKI therapy, and the impact of the uncommon mutations found in Asian patients is unknown (17, 18). Clinical studies so far have focused on the TKI treatment of NSCLC BMs with sensitizing mutations. Gefitinib is indicated in the treatment of EGFR-positive NSCLC BM and erlotinib as the second-line treatment for BM from asymptomatic NSCLC (1). The BRAIN trial (CTONG1201) showed that icotinib significantly improved the progression-free survival (PFS) and intracranial objective response rate (ORR) of patients with *EGFR* mutation and BMs (19). The ongoing APOLLO trial (ClinicalTrials.org #NCT02972333) is examining the efficiency and safety of osimertinib EGFR TKI in the treatment of *EGFR* mutated patients with BMs. Based on the *post hoc* analysis of the LUX-Lung 2/3/6 trials (9, 20, 21), the treatment indication for afatinib has been expanded to the first-line treatment of metastatic NSCLC with non-resistant *EGFR* mutation including L861Q/G719X/S768I. Afatinib is able to cross the BBB in sufficient amounts to induce anti-tumor actions (22, 23).

Several studies showed that *EGFR* mutation patterns in NSCLC primary lesions and metastases in various body locations are not consistent with that found in the BMs (24–26), possibly because of the specific events required for cancer cell migration to and survival in the brain. Indeed, a primary tumor is composed of various clones (27, 28) and not all of them will have the abilities to spread in circulation, cross the BBB, survive in the brain microenvironment, and invade the brain tissue (1, 29). These abilities call for specific sets of factors and mutations and therefore the actual tumor mutation status of BMs may differ from the estimation using primary tumor tissue or peripheral blood (12, 30). Indeed, a discordance rate of 16–32% for *EGFR* mutation status (depending on assay sensitivity for mutational analysis) between the primary site and BMs has been previously reported (12). Recent studies indicated that cerebrospinal fluid (CSF) ctDNA from BMs were present in CSF and that clinically actionable *EGFR* mutations were also more frequently detected in CSF ctDNA than in plasma in patients with BMs (31). Therefore, there is a possibility that BMs harboring rare mutations (e.g., L861Q, G719X, and S768I) not found in the primary lesion or metastases in other body locations will respond to EGFR-TKIs that are effective against lesions harboring those rare mutations, e.g., afatinib (9, 20, 21).

Therefore, EGFR-TKI can be used for the management of BMs from NSCLC, but the significance of uncommon *EGFR* mutations on the development and treatment response of BMs is still unclear. There are no studies on the significance of uncommon *EGFR* mutations in patients with BMs from NSCLC. We hypothesized that EGFR-TKIs could be effective against BMs with uncommon *EGFR* mutations, as evaluated by CSF ctDNA. The objectives of the present study were: (1) to evaluate the *EGFR* mutations in patients with NSCLC and newly diagnosed BMs; and (2) to examine the effect of EGFR-TKI on BMs harboring uncommon *EGFR* mutations.

## Methods

### Study Design and Patients

This was a prospective study of 21 consecutive patients with NSCLC and BMs diagnosed between April 2018 and January 2019. The study was approved by the ethics committee of Tianjin Huanhu Hospital. All patients provided written informed consent prior to any study procedure. The inclusion criteria were: (1) NSCLC confirmed by histopathological examination; (2) new diagnosis of BMs by MRI and CSF cytological test with ThinPrep [a liquid-based cytology test applied in the diagnosis of LM (29)]; and (3) no prior treatment against BMs.

### Data Collection

Demographics, clinical data, pathological data, imaging data, and tumor markers [carcinoembryonic antigen (CEA)] were obtained routinely. The *EGFR* mutation status of the primary site was obtained from previous medical records.

### Samples, DNA Extraction, and Next-Generation Sequencing

CSF samples were obtained from all 21 patients by lumbar puncture and placed in SanMed fixative solution, a patented cell preservation solution (Zhuhau SanMed Diagnostics Inc.), for transport and storage. Total DNA was extracted from CSF using the QiAamp Circumstance Nucleic Acid kit (#55114, Qiagen, Venlo, The Netherlands) according to the manufacturer's instructions. The reference library was constructed using the Ion AmpliSeq Library Kit 2.0 and the Ion AmpliSeq Cancer HosSpot Panel v2 (#55114 and #4475346, Thermo Fisher Scientific, Waltham, MA, USA) and the Ion Library TaqMan Quantitation kit (#4468802, Thermo Fisher Scientific, Waltham, MA, USA), according to the manufacturer's instructions. Details on next-generation sequencing are provided in Supplementary File 1.

### Statistical Analysis

Due to the relatively small sample size, only descriptive statistics were used. Data are presented as numbers and percentages.

## Results

### Characteristics of the Patients

Among the 21 patients with NSCLC, there were 10 (47.6%) males and 11 (52.4%) females. The mean age was 59.7 ± 9.9 years. Ten (47.6%) patients had LMs, while 11 (52.4%) had PBMs. Thirteen (61.9%) patients had primary tumors confirmed with *EGFR* mutation.

### Uncommon *EGFR* Mutations

**Figure 1:**
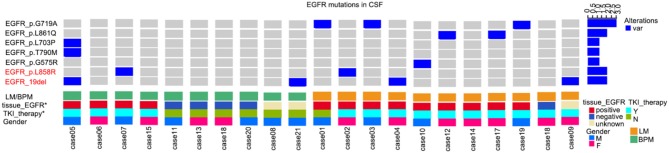
Uncommon mutations in the *epidermal growth factor receptor* (*EGFR*) gene from cerebrospinal fluid (CSF) circulating tumor DNA (ctDNA) from patients with non-small cell lung cancer (NSCLC). BPM, brain parenchymal metastases; LM, leptomeningeal metastases; TKI, tyrosine kinase inhibitor.

The uncommon mutation-positive rate in CSF ctDNA from all study subjects was 33.3% (7/21) (Figure 1). Among the patients with primary tumors with *EGFR* mutation, the rate of uncommon mutations was 53.8% (7/13). Six of these seven patients were treated with TKI and showed disease progression in the brain during the course of treatment.

Compared with wild type *EGFR*, patients with primary tumors with *EGFR* mutation were more likely to display an uncommon *EGFR* mutation in CSF ctDNA (7/13, 50% vs. 0/5, 0%). Uncommon mutations were also more common in patients with LM than in patients with PBM (6/11, 54.5% vs. 1/10, 10%).

### Effectiveness of EGFR TKI in Patients With Uncommon Mutations in CSF ctDNA

For the seven patients with uncommon *EGFR* mutations in CSF ctDNA (regardless of *EGFR* mutations status in brain/lung tissues), TKI was effective in four cases (57.1%), as shown by MRI and CEA levels.

**Figure 2:**
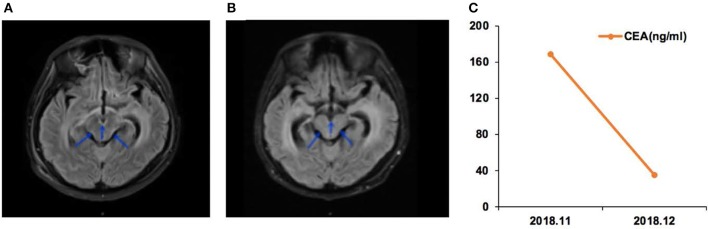
Case 01 was a male of 34 years of age, with lung adenocarcinoma and with a history of smoking, but quitted 10 years ago. **(A)** T2 FLAIR enhanced magnetic resonance imaging (MRI) showed abnormal high signal in the medulla, oblongata, pon, and ventral and dorsal midbrain, suggesting leptomeningeal metastases (LMs). **(B)** T2 FLAIR enhanced MRI during afatinib treatment showed that the abnormal high signal in the medulla, oblongata, and ventral and dorsal midbrain was lower than before treatment. **(C)** Carcinoembryonic antigen (CEA) levels before and after afatinib treatment.

Case 01 was a male of 34 years of age, with lung adenocarcinoma and with a history of smoking, but quitted 10 years ago (Figure 2). In April 2018, LM was diagnosed, and the *EGFR* p.G719A mutation was detected in CSF ctDNA (55.6%). The CSF CEA level was 9,470 ng/ml. The patient started afatinib treatment in May 2018, and achieved a partial response by July 2018, with a CSF CEA level of 2,111 ng/ml. The response was maintained in November 2018, with a CSF CEA level of 1,590 ng/ml and CSF *EGFR* p.G719A mutation at 23.1%.

**Figure 3:**
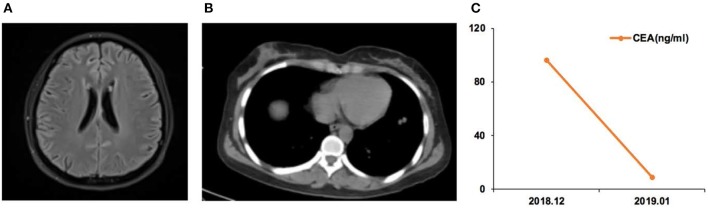
Case 05 was a male of 71 years of age, with lung adenocarcinoma but without smoking history. **(A)** Cerebellar vermis, bilateral cerebral hemispheres, and pia meninges shoed abnormal enhancement on magnetic resonance imaging. Leptomeningeal metastasis (LM) was considered. **(B)** Chest computed tomography revealing the primary lung lesion. **(C)** Carcinoembryonic antigen (CEA) levels before and after osimertinib treatment.

Case 05 was a male of 71 years of age, with lung adenocarcinoma but without smoking history (Figure 3). The *EGFR* 19Del mutation was detected in the primary tumor. He received oral icotinib for 8 months before being admitted to the hospital for dizziness and episodes of loss of consciousness and was diagnosed with PBM. In December 2018, the *EGFR* p.L703P (2.0%) and *EGFR* p.T790M (2.1%) mutations, and the *EGFR* 19Del (86.0%) were detected in CSF ctDNA. The CSF CEA level was 96.1 ng/ml. The patient started osimertinib (80 mg qd) treatment, and the neurological symptoms were alleviated. In January 2019, the CSF CEA level was 8.7 ng/ml.

**Figure 4:**
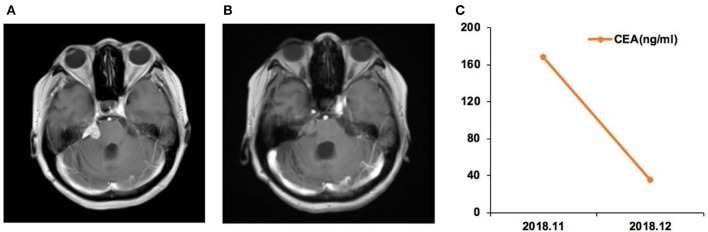
Case 12 was a female of 57 years of age, with lung adenocarcinoma but without smoking history. **(A)** In September 2018, the right cerebellopontine angle area, the edge of the tetras, and the lateral edge of the right arm were abnormally enhanced on magnetic resonance imaging. **(B)** In December, the enhancement intensity was decreased on the right side, and her condition was improved. **(C)** Carcinoembryonic antigen (CEA) levels before and after afatinib treatment.

Case 12 was a female of 57 years of age, with lung adenocarcinoma but without smoking history (Figure 4). She was diagnosed with LM in September 2018. CSF ctDNA analysis revealed the *EGFR* p.L861Q (46.5%) mutation, and the CSF CEA level was 786.9 ng/ml. She started afatinib treatment. In December 2018, the CSF CEA level was 98.1 ng/ml.

**Figure 5:**
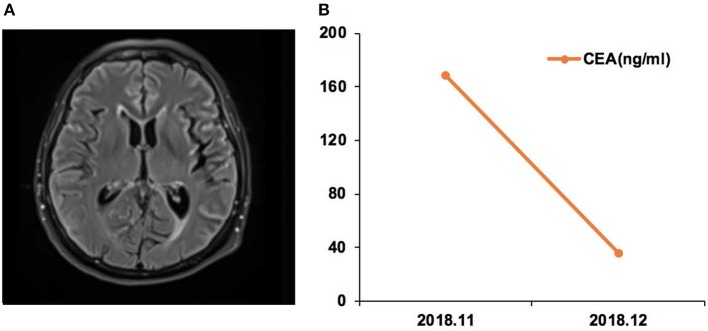
Case 17 was a female of 65 years of age, with lung adenocarcinoma but without smoking history. **(A)** Magnetic resonance imaging (MRI) of the brain. **(B)** Carcinoembryonic antigen (CEA) levels before and after afatinib treatment.

Case 17 was a female of 65 years of age, with lung adenocarcinoma but without smoking history (Figure 5). In November 2018, CSF ctDNA analysis revealed *EGFR* p.L861Q (62.6%) and TP53 p.C135F (95.5%) mutations, and the CSF CEA level was 168.3 ng/ml. The patient started afatinib treatment. In December 2018, the CSF CEA level was 35.4 ng/ml.

## Discussion

The rate of uncommon *EGFR* mutations in Asian patients with NSCLC is high, comprising 11.9% of all cases in a previous report (31). There were rare previous studies on the significance of *EGFR* uncommon mutations in patients with NSCLC and BMs. There is a possibility that BMs harboring rare mutations not found in other body locations will respond to EGFR-TKIs (9, 20, 21). Therefore, the aim of this study was to evaluate the *EGFR* mutations in patients with NSCLC and newly diagnosed BMs and examine the effect of EGFR TKI on BMs harboring uncommon *EGFR* mutations. The results showed that the rate of uncommon *EGFR* mutation in patients with NSCLC and BMs was high. The BMs with uncommon *EGFR* mutations seemed to respond to EGFR TKI treatment. Taken together, CSF liquid biopsy could reveal the *EGFR* genetic profile of the BM and help guide treatment using small-molecule TKI. These results do not imply that metastases in other body locations will answer or not to the BM-guided therapy, but since survival to BMs is short (2, 3), tailoring EGFR-TKI treatment specifically to the BMs might has a higher likelihood of prolonging survival in those patients.

In this study, the frequency of uncommon *EGFR* mutations was high, with these mutations detected in the CSF ctDNA in 33.3% (7/21) patients (considered to be from the BMs). These rates are higher than the 12% previously reported in patients with NSCLC but not necessarily with BM in China (16). This discrepancy might be due to the small sample size (selection bias) and the different testing methods. On the other hand, *EGFR* mutations have been reported to be more frequent in patients with NSCLC and BM (32). The exact role of uncommon *EGFR* mutations in BM development requires further research.

A primary tumor is a mosaic of various clones that evolved from the original tumor cell(s) (27, 28). Unlike cytotoxic chemotherapies that target all fast-growing cells, targeted treatments target specific cells within the tumor, raising the possibility of selecting resistant or unaffected clones, which can be responsible for relapse and metastasis (33, 34). BMs show significant molecular divergence with the primary tumor and with extracranial metastases (30, 31, 35–39). The process of BM development from the primary tumor necessitates specific steps, including crossing the BBB, surviving in the brain microenvironment, and invading the brain tissue, all of which requiring specific sets of biological aspects (1). The development of BMs in lung cancer patients who received an anti-EGFR treatment may be due to the TKI effectively killing the cancer cells with the exon 19 deletion or the L858R mutation, but the effect of the TKI could be insufficient on the cells with uncommon mutation, therefore increasing the possibility of these cells contributing to BM development. Indeed, it has been shown that mutations such as exon 20 insertions, L861Q, S768I, and G718X have inferior response to first- generation EGFR TKIs (40). In the present study, six of the seven patients with BMs harboring uncommon *EGFR* mutations had received adjuvant EGFR TKI, supporting the hypothesis of clone selection by EGFR TKI. Nevertheless, additional studies are necessary to examine this point since erlotinib has been shown to reduce the risk of BMs from NSCLC (41).

A number of studies indicated the efficacy of EGFR TKI treatment against NSCLC BMs (1, 26, 42). The results from the LUX-Lung 2/3/6 trials (9, 20, 21) indicate that afatinib can be used as first-line treatment of metastatic NSCLC with non-resistant EGFR mutation including L861Q/G719X/S768I. Of particular interest, afatinib is able to cross the BBB in sufficient amounts to induce anti-tumor actions (22, 23). In the present study, three patients with uncommon *EGFR* mutations responded well to afatinib, as shown by MRI and CEA levels. A good response was also observed with Osimertinib. Additional studies are necessary to determine the best treatment approaches for BMs harboring uncommon mutations, particularly in the context that the frequency of those mutations is high in Asia (16).

Obtaining genetic material from BMs is complicated because surgical resection and biopsy are often impossible or not indicated due to the patient's condition. The BBB prevents ctDNA from brain lesions to pass into the blood circulation and vice versa; therefore, the ctDNA found in CSF by liquid biopsy will reflect the status of the BMs (38, 43–46). Hence, a liquid biopsy of CSF in patients with NSCLC and BMs could provide the actual intracranial situation, helping to guide patient management. New technologies such as next-generation sequencing will allow personalized medicine to reach its full potential (38, 44).

It is well-known that LMs are less common than PBM, but their prognosis is poorer (4, 5). In the present study, the frequencies of LMs and PBMs were similar, hinting toward some possible selection bias. Nevertheless, an important result is that the frequency of uncommon *EGFR* mutation was higher in LMs than in PBMs. This could explain, at least in part, the poorer prognosis of LMs. The association of uncommon *EGFR* mutation and LM will have to be examined in future studies.

The present study had limitations. Because uncommon mutations are rarely diagnosed, the sample size was relatively small, and the study was performed in a single center. In addition, follow-up was short. Furthermore, no post-treatment radiological data were available in some cases after patient improvement and discharge, especially non-residents. Moreover, CEA assessment is not widely accepted as a response marker. Finally, patients were administered various TKIs that had different BBB penetration rates.

## Conclusions

EGFR TKI could be effective against uncommon *EGFR* mutations in NSCLC BMs. Molecular testing of CSF could be helpful in guiding treatment and tracking treatment response. Uncommon mutation might be considered as participating in the process of brain metastases of NSCLC.

## Data Availability Statement

The datasets used and/or analyzed during the current study are available from the corresponding author on reasonable request.

## Ethics Statement

The studies involving human participants were reviewed and approved by the ethics committee of Tianjin Huanhu Hospital. The patients/participants provided their written informed consent to participate in this study.

## Author Contributions

CM, CH, RJ, and JZ conceived and coordinated the study, designed, performed, analyzed the experiments, and wrote the paper. JL, NM, ZL, and LX carried out the data collection, data analysis, and revised the paper. All authors reviewed the results and approved the final version of the manuscript.

### Conflict of Interest

JZ, CH, DT, XY, and LX were employed by Zuhai SanMed Biotech Ltd. RL and ZL were employed by Zuhai Livzon Gene Diagnostics Ltd. The remaining authors declare that the research was conducted in the absence of any commercial or financial relationships that could be construed as a potential conflict of interest.

## Supplementary Material

Supplementary File 1 Details of next-generation sequencing.

## Abbreviations

BBB: blood-brain barrier
BM: brain metastases
CEA: carcinoembryonic antigen
CSF: cerebrospinal fluid
*EGFR*: *epidermal growth factor receptor*
LMs: leptomeningeal metastases
NSCLC: non-small cell lung cancer
ORR: objective response rate
PBMs: parenchymal BMs
PFS: progression-free survival
TKI: tyrosine kinase inhibitors.
