# Supplementary material for: Tyrosine Kinase Inhibitors Could Be Effective Against Non-small Cell Lung Cancer Brain Metastases Harboring Uncommon EGFR Mutations
Source: Front Oncol. 2020 Mar 5;10:224. doi: 10.3389/fonc.2020.00224 (PMC7066117; doi:10.3389/fonc.2020.00224)
Supplement: Supplementary File 1 — Details of next-generation sequencing. [file Table_1.DOCX]

**PCR amplification and sequencing**

PCR amplification was performed using either a Veriti Dx 96-well Thermal Cycler (#299121427, Thermo Fisher Scientific, Waltham, MA, USA), ProFlex PCR System 3×32 well (#2978018082204, Thermo Fisher Scientific, Waltham, MA, USA), Veriti 60-well (#299040425, Applied Biosystems, Foster City, CA, USA), or Veriti 96-well (#299121194, Applied Biosystems, Foster City, CA, USA).

Blood CTCs were analyzed using the Ion 540 Kit (#A27759, A30011, and A27766, Thermo Fisher Scientific, Waltham, MA, USA), according to the manufacturer's instructions. CSF ctDNA and tissue DNA were analyzed using the Ion 540 Chip (#27766), Ion 510/520/530 Kit-Chef 2R/I (#A34461), Ion 530 Chip Kit (#A27764), Ion PI Chip Kit v3 (#A26771), Ion PI HIQ Seq 200 Kit (#26772), and Ion PI HI-Q Template OT2 200 (#A26434) (Thermo Fisher Scientific, Waltham, MA, USA), according to the manufacturer's instructions. The Ion analysis was performed was performed using the Ion Chef System (#242470686, Thermo Fisher Scientific, Waltham, MA, USA), Ion One Touch ES (#410122, Thermo Fisher Scientific, Waltham, MA, USA), and Ion One Touch 2 (#2456280-0935, Thermo Fisher Scientific, Waltham, MA, USA) for sample preparation, and Ion Torrent S5XL Sequencer (#2772817040192, Thermo Fisher Scientific, Waltham, MA, USA), for sequencing.
